# Two of a Kind or a Full House? Reproductive Suppression and Alloparenting in Laboratory Mice

**DOI:** 10.1371/journal.pone.0154966

**Published:** 2016-05-05

**Authors:** Joseph P. Garner, Brianna N. Gaskill, Kathleen R. Pritchett-Corning

**Affiliations:** 1 Stanford University, Department of Comparative Medicine, and by courtesy, Department of Psychiatry and Behavioral Sciences, Stanford, California, United States of America; 2 Charles River, Wilmington, Massachusetts, United States of America; 3 Purdue University Department of Comparative Pathobiology, West Lafayette, Indiana, United States of America; 4 Harvard University Faculty of Arts and Sciences, Office of Animal Resources, Cambridge, Massachusetts, United States of America; University of Tasmania, AUSTRALIA

## Abstract

Alloparenting, a behavior in which individuals other than the actual parents act in a parental role, is seen in many mammals, including house mice. In wild house mice, alloparental care is only seen when familiar sibling females simultaneously immigrate to a male’s territory, so in the laboratory, when a pair of unfamiliar female wild mice are mated with a male, alloparenting does not occur because one female will typically be reproductively suppressed. In contrast, laboratory mice are assumed to alloparent regardless of familiarity or relatedness and are therefore routinely trio bred to increase productivity. Empirical evidence supporting the presence of alloparental care in laboratory mice is lacking. Albino and pigmented inbred mice of the strain C57BL/6NCrl (B6) and outbred mice of the stock Crl:CF1 (CF1) were used to investigate alloparenting in laboratory mice since by mating pigmented and albino females with albino males of the same stock or strain, maternal parentage was easily determined. We housed pairs (M:F) or trios (M:2F) of mice in individually ventilated cages containing nesting material and followed reproductive performance for 16 weeks. Females in trios were tested to determine dominance at the start of the experiment, and again 5 days after the birth of a litter to determine if a female’s dominance shifted with the birth of pups. Results showed a significant and expected difference in number of offspring produced by B6 and CF1 (p < 0.0001). Pigmented mice nursed and nested with albino pups and vice-versa, confirming empirical observations from many that group nesting and alloparenting occurs in unrelated laboratory mice. When overall production of both individual mice and cages was examined, reproductive suppression was seen in trio cages. Dominance testing with the tube test did not correlate female reproduction with female dominance in a female-female dyad. Due to the reproductive suppression noted in trios, on a per-mouse basis, pair mating outperformed trio mating (p = 0.02) when the measure was weaned pups/female/week. No infanticide was seen in any cages, so the mechanism of reproductive suppression in trio matings may occur before birth.

## Introduction

Alloparenting is defined as individuals other than the biological parents acting in a parental role to young animals. Since parental roles vary by sex and time, alloparenting may include provisioning of offspring, defense of offspring, or both [1]. Many mammals exhibit this behavior, which is thought to increase overall offspring survival. Alloparenting has been observed in animals as diverse as callitrichid monkeys [2], voles [3], gerbils [4],and foxes [5]. Alloparenting usually occurs where there is some benefit to both parties; this benefit could be related to kin selection or some mutually beneficial reciprocation. Kin selection theory posits that animals will, on average, aid related animals before unrelated animals in order to propagate copies of their genes present in related individuals [6]. According to kin selection theory, animals would be more likely to alloparent their full sibling’s offspring, but might kill the offspring of an unrelated animal [7]. The cost-benefit analysis of the animals involved provides additional reasons for alloparenting beyond kin selection. For example, in mice, the benefit to the alloparent may be access to the communal nest, thus gaining warmth and shelter. Alloparenting may improve the quality of the care and support given to offspring by increasing the fitness of the parents [8, 9]. This might be achieved by decreasing the energetic demands on lactating females or by allowing parents to leave the offspring to patrol territory and repel intruders [10].

While alloparental care is seen in some rodents, as noted above, the case for it being a widespread behavioral strategy in wild and domestic mice is less certain. As part of their development, juvenile male mice generally disperse from their natal territory to attempt to establish their own territory [11]. Females also emigrate from their natal territory to find mates. In wild house mice, alloparental care is more likely to be seen when familiar sibling females simultaneously immigrate to a male’s territory [12]. These females will reproduce at the same time, nest communally, and nurse each other’s pups. Two conditions must be met in wild mice for alloparenting to occur: familiarity as well as relatedness, reinforcing the kin selection benefit theory noted above [7, 13, 14]. When unfamiliar female wild mice are introduced to a male in the laboratory, one female will fail to wean offspring through reproductive suppression or infanticide [12, 15]. Although extensively studied in wild mice both in the field and in the laboratory, few studies on alloparenting have been performed on laboratory mice in typical mating configurations.

Mice have generally adapted well to domestication and the laboratory as well as to management of their breeding by scientists. This behavioral and genetic plasticity, combined with the constraints of laboratory housing and husbandry, has led to the common practice of mating mice in various configurations of males and females to maximize production in the often limited space of animal housing facilities. In addition, laboratory mice have long been thought to alloparent regardless of familiarity or relatedness. Laboratory mice are typically a mix of *M*. *m*. *musculus*, *M*. *m*. *domesticus*, and *M*. *m*. *castaneus*, often with genetic contributions from other species such as *M*. *spretus* [16] and they descend from mice domesticated thousands of years ago for various purposes, including ceremonial or sacred purposes and as pets [17]. Laboratory mice are typically broadly classified as either inbred or outbred, also known respectively as strains and stocks. Inbred strains of mice originate from a single pair and pedigreed animals are maintained strictly through brother/sister matings. Outbred stocks are maintained to maximize genetic diversity in a closed colony and brother/sister and cousin matings are avoided as much as possible. Although inbred mice must be mated in pairs (M:F) for pedigree purposes, a more common mating scheme is to mate mice in trios of a male and 2 females (M:2F) if pedigree is not important. Trio mating is assumed to maximize production and increase pup survival due to alloparenting.

However, because pairs of female laboratory mice set up in trios are rarely sisters, alloparenting may not be occurring because although there is simultaneous “immigration” to the male’s territory, the criterion of relatedness is not also met. In laboratory mice, the conditions under which alloparenting occurs have never been formally tested. Therefore, we used a novel application of simple coat color genetics as a method of determining parentage in laboratory mice to test this hypothesis. To test for reproductive suppression we recorded measures of breeding performance. As additional measures of potential stress or poor welfare within the cages, we also examined wounding, alopecia [18], and infanticide [12].

## Materials and Methods

All work was conducted at Charles River’s AAALAC-accredited Wilmington, MA, facility and was approved by Charles River’s Institutional Animal Care and Use Committee (P04022012). At the start of study, animals were free of a list of common mouse infectious agents; further details may be found at http://www.criver.com/files/pdfs/rms/hmsummary.aspx. No further health monitoring was performed. Animals used in this study were surplus stock from standard breeding programs that were redirected to this study. Albino and pigmented inbred C57BL/6NCrl (B6) and outbred Crl:CF1 (CF1) were used. All pigmented B6 mice were black, but since pigmented CF1 were the result of breeding a spontaneous reversion mutation of the tyrosinase gene (*Tyr*) arising at Charles River, various colors of CF1 were seen, although animals used in this study were agouti or brown. For both the B6 and CF1 mice, 8 albino males, 6 pigmented females, and 6 albino females began the study for a total of 16 males and 24 females or of 40 animals. Since the albino allele of the *Tyr* gene is recessive to the wild-type allele that results in pigment production, by mating pigmented and albino females with albino males of the same stock or strain, maternal parentage of pups was easily determined because pups are the same color as their dam. Females were approximately 49 days of age (+ 3d) at study start, while males were approximately 56 days of age (+ 3d). We housed pairs (M:F) or trios (M:2F) of mice in disposable individually ventilated cages (Innovive, San Diego, CA). All cages were bedded with heat-treated shredded aspen bedding (NEPCO, Warrensburg, NY), and mice were provided with 8–10 g of a long-fiber paper nesting material (EnviroDri, Shepherd Specialty Papers, Watertown, TN) at the weekly cage change. Food (5L79, LabDiet, St. Louis, MO) and ultrafiltered hyperchlorinated water (via water bottle) were provided *ad libitum*. The light cycle was 12:12 light:dark (on at 0630, off at 1830), humidity was maintained between 30–70%, and temperature ranged between 19–22°C. The cage was the experimental unit, so if an adult in a cage became ill, the entire cage was euthanized via inhalation of CO_2_ and the cage replaced with the same experimental unit. At the end of the study, all animals were euthanized with CO_2_ inhaled to effect.

**Fig 1:**
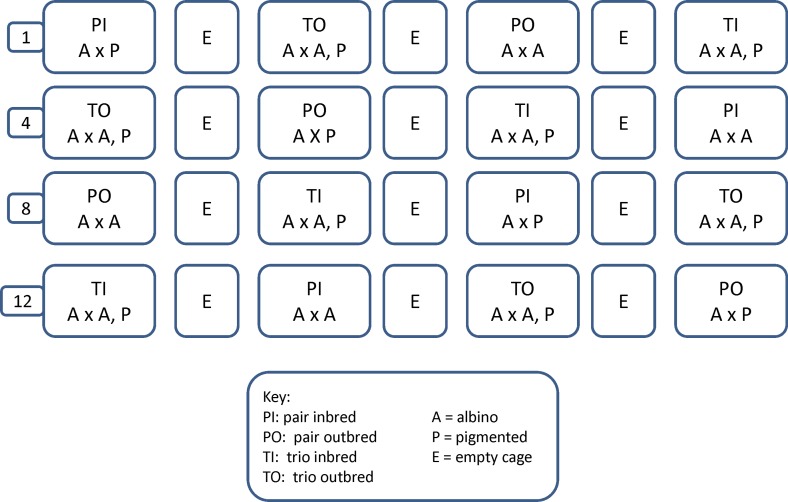
Diagram of cage location on the rack. The figure illustrates the location of all the cages followed in this experiment. C57BL/6 mice are identified as inbred and CF1 mice as outbred. Number on the left hand side of the diagram indicates the row of the rack the cage was placed on.

Breeding cages were balanced across rows of the housing rack (Fig 1). Females were assigned to breeding condition (pair or trio) based on coat color using the random integer set generator found at random.org. Data on reproduction were collected for 16 weeks. Data collected included: female parent of litter born, date of birth of pups, number of pups born, date of wean of pups, number of pups weaned, sex of pups weaned, pup weaning weight, and the presence or absence of infanticide, defined as litters born and disappearing, finding dead pups, finding parts of pups, or observing animals killing pups. The production index (PI) was calculated as the number of pups weaned per female per week. This was calculated on a whole cage basis as well as on an individual female basis for trio cages. Survivorship was calculated as the number of pups weaned divided by the total number of pups born, whether dead or alive. Sex ratio of pups born was also calculated from the reproductive data collected above. Additional data collected to assess conditions in the cage included nest score, alopecia score of pups, and wounding score of adults (SI 1), all collected weekly at cage change while lights were on, between 1-3pm on Monday. Cages were examined for alloparenting when nest scoring was conducted. Alloparenting was scored as a yes/no measure by examining nests for the presence of pups of both colors when more than one litter was present in the cage and scored as yes when pups of a different color were seen nursing a dam. Females in trios were dominance tested in the animal housing room using a standard test to determine dominance (the tube test originally described by Lindzey [19] and modified by Howerton [20]) at the start of the experiment, and also when their pups were 5 days old to determine whether a female’s dominance shifted with the birth of pups.

We used a repeated measures Latin square design (2 types of mice x 2 pigments x 2 breeding configurations x 2 replicates = 16 cages), a design specifically used to maximize power and reduce sample size because it incorporates, accounts for, and eliminates unwanted variance. The use of factorial and repeated measures design is commonly used as a means of reduction and refinement. This approach allows us to test for general effects of each variable, and also look for interactive effects with greater power than non-factorial analyses [21]. Power cannot be directly calculated for complex factorial designs [22]. Cage is the experimental unit in this experiment and all data are expressed as cage averages.

Data were analyzed as a GLM in JMP (SAS, Cary, NC). All cage level analyses were run in a simple two-way factorial design in GLM, testing for the effects of treatment, strain, and their interaction. All individual female analyses were run as a two-way factorial nested block design in GLM. Cage, nested within treatment and strain, was included as a blocking factor, testing for the effects of treatment and strain, and their interaction. Cage was considered to be a fixed blocking factor, as the true random effect component of cage (i.e. the mice), are represented by the residual. The effect of dominance on trio breeding performance was only examined in trios, and thus employed a nested block design in GLM. Cage nested within strain was included as a blocking factor. Strain, mean dominance score, and their interaction were tested. For the same reasons as above, cage was treated as a fixed effect. The blocking factor pigment status did not significantly explain any of the data and therefore was not included in any of the analyses. Pup alopecia data, for both cage level and individual female levels, was log transformed for normality. The assumptions of GLM (normality of error, homogeneity of variance, and linearity) were confirmed post-hoc, and appropriate transformations were made to meet these assumptions [21]. Significant effects were then analyzed using post-hoc Tukey tests. All values are given as least squares means and standard errors. All data are available as Supplemental Information (S1 Data).

To determine if a reproductive bias occurred in trio mated groups, we calculated the bias in the 8 trios as the percent of pups weaned to the female producing the least pups. We then used the standard Z transformation of the proportion and tested it against an expected value of 50%. This is widely used in birth ratio literature to convert ratios into both a normally distributed variable, and to scale that variable by the number of individuals (pups) involved, e.g. Thogerson [23]. We then used these Z scores in a simple GLM to test whether there was overall evidence for reproductive suppression, and whether this differed by strain.

## Results

**Fig 2:**
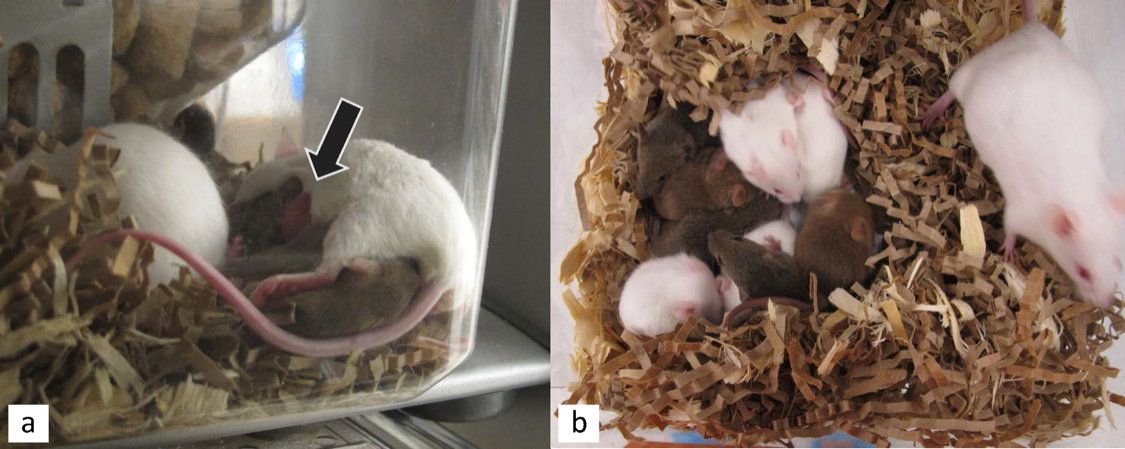
**Photographs of mice alloparenting**: a) A female albino mouse is seen nursing several of the pigmented female’s pups. The black arrow indicates a newborn pup also nursing at the same time as the older pigmented pups. b) A communal nest site containing pups from both the albino and pigmented female.

Two adult CF1 females from separate trio cages and three male B6 from two trio cages and one pair cage were euthanized, the females for dystocia and the males for paraphimosis. This increased the total number of animals used from 40 to 54, since entire cages were replaced, but the number of animals analyzed remained the same (40). No wounding was seen in any mice. Alloparenting, when pups not belonging to a dam were observed suckling was seen in 5 of 8 trio cages (Fig 2A). In one trio cage, one female did not have any litters during the experiment and therefore alloparenting was not observed. In the other two cages, one female did not reproduce until the very end of the experiment, therefore limiting the amount of time the females would be able to alloparent or to be observed alloparenting. When two litters were present at the same time, pups of both colors were found together in one nest (Fig 2B). Alloparenting was observed in all trio cages where both females had litters simultaneously present in the cage for long enough that observation could occur. Pups of various ages from newborn to weanlings could be found in the nest. Although females without litters were occasionally observed in separate nests, if a litter was in the cage, there was one communal nest.

### Data analyzed per individual female

**Fig 3:**
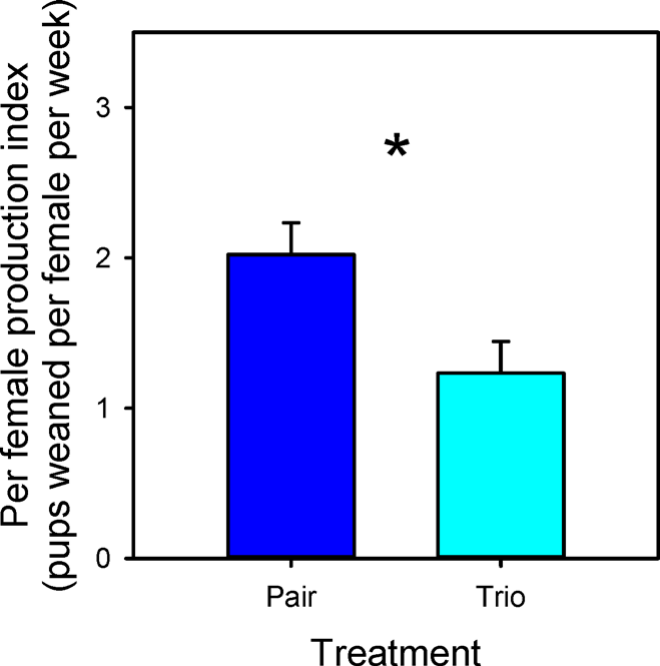
Per female production index: LSM and SE values for pair and trio breeding treatments are plotted along the x axis. * Indicates a significant difference (P < 0.05) between the two values.

**Fig 4:**
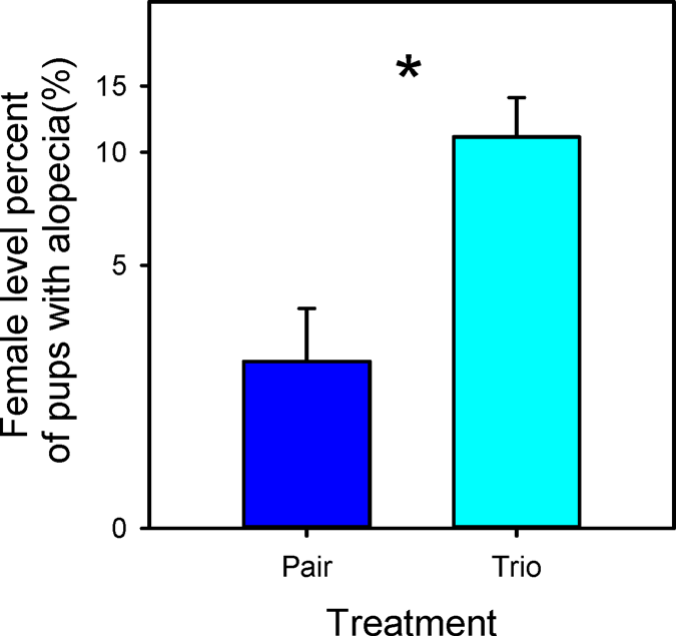
Percentage of pup alopecia (hair loss) averaged per female: LSM and SE values for pair and trio breeding treatments are plotted along the x-axis. Percentages are plotted along a log-transformed y-axis. * Indicates a significant difference (P < 0.05) between the two values.

When the production index (PI; pups weaned/female/week) was examined, pair mating outperformed trio mating on a per-female basis (F_1,16_ = 6.5; P = 0.02; Fig 3). As expected, and also on a per female basis, outbred CF1 females had a higher PI than inbred B6 (CF1 2.4 ± 0.22; B6 0.8 ± 0.22; F_1,16_ = 25.9; P < 0.001). There was no statistical difference in survivorship on average for an individual female’s litter based on any of the treatments. As in the cage level analysis, pups from CF1 females had a greater weaning weight than those from B6 females (CF1 13.2 ± 0.63; B6 8.8 ± 0.65; F_1,15_ = 50.3; P < 0.001). Pup alopecia was increased in trios compared to pairs (F_1,7_ = 11.6; P = 0.011), (Fig 4) but no strain effect was seen (F_1,7_ = 3.2; P = 0.11). Sex ratio did not differ based on any of the treatments or their interactions. An individual female’s average performance on a standard test of dominance was not related to her reproductive output in a trio mating (F_1,6_ = 0.6; P = 0.44).

### Data analyzed on a cage level basis

**Fig 5:**
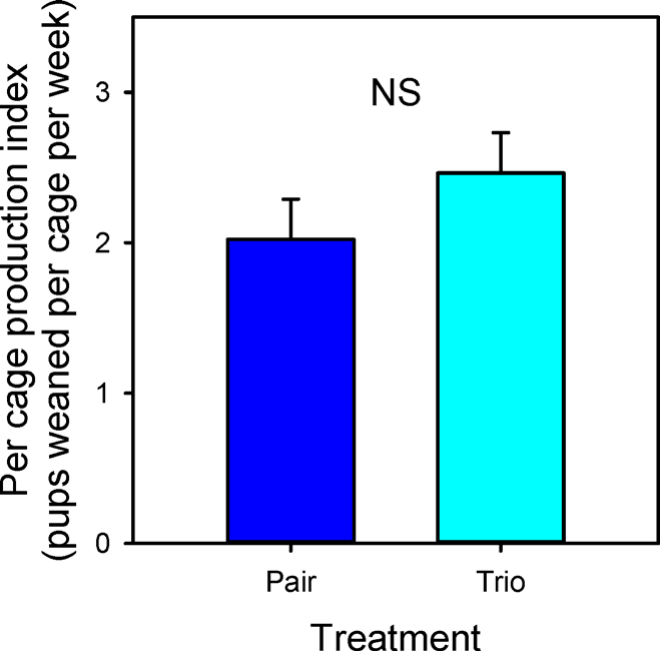
Cage average production index: LSM and SE values for pair and trio breeding treatments are plotted along the x axis. NS indicates there is no significant difference between the two values.

**Fig 6:**
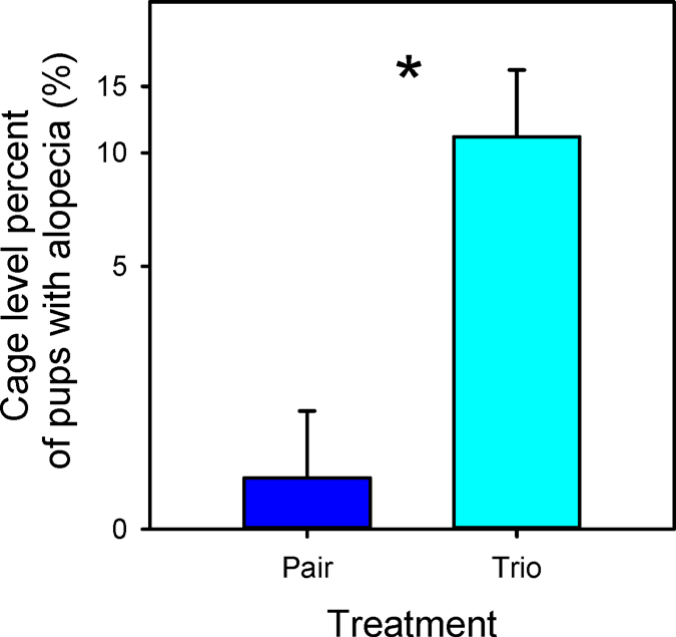
Percentage of pup alopecia (hair loss) averaged per cage: LSM and SE values for pair and trio breeding treatments are plotted along the x-axis. Percentages are plotted along a log-transformed y-axis. * Indicates a significant difference (P < 0.05) between the two values.

CF1 cages had a higher PI than B6 cages (3.32 ± 0.27 for CF1, 1.16 ±0.27 for B6; F_1,12_ = 32.6; P < 0.001). Although trio matings had a slightly higher PI than pairs, the difference was not statistically significant (trio matings 2.5 ± 0.27; pair matings 2.0 ± 0.27; F_1,12_ = 1.3; P = 0.27; Fig 5). Infanticide, defined as finding dead pups, finding parts of pups, or observing animals killing pups, was not observed in any cage. However, 100% survivorship of pups was only seen in 2 cages (1 cage of paired CF1 and 1 cage of paired B6. Survivorship was not altered at the cage level by any of the treatments or interactions. CF1 pups had a higher weaning weight than did B6 pups (CF1 13.1 ± 0.35, B6 8.8 ± 0.35; F_1,12_ = 76.0; P = 0.004). No strain effect in alopecia percentage was seen (F_1,12_ = 13.0; P = 0.33), but pups from trio mating cages had a higher percentage of alopecia (F_1,12_ = 13.0; P = 0.003; Fig 6) Sex ratio of pups was not different between pair and trio mated cages (F_1,12_ = 0.17; P = 0.7). Paired mice also had higher nest scores than trios (trio matings 3.8 ± 0.09; pair matings 4.2 ± 0.09; F_1,12_ = 12.2; P = 0.04) and B6 mice had higher nest scores than CF1 (CF1 3.8 ± 0.09; B6 4.3 ± 0.09; F_1,12_ = 13.4; P = 0.003). Six of 8 trios had highly significant biases (i.e. pup birth ratios where one female was significantly reproductively suppressed). Overall, trios consistently showed significant reproductive suppression of one female so that mean bias was significantly different from 50/50 (T_6_ = -3.18; P = 0.0190), but the strains did not differ significantly in their mean bias (F_1,6_ = 0.0001; P = 0.9926).

## Discussion

Alloparenting has been described in wild mice, but only in very limited social contexts [12–15]. The limited social contexts under which wild mice will alloparent do not typically reflect the way in which we breed laboratory mice. In this paper, we sought to use laboratory mice to test if alloparenting occurred in these domesticated animals in their typical artificial social milieu. Our key findings are that: 1) alloparenting did occur in all trio-mated cages; 2) reproductive suppression of one female occurred in the majority of trio-mated cages. In other words, laboratory mice that are trio-mated behave both unlike and like their wild counterparts.

In this study, females and males were placed into the test cage at the same time, although females originated from separate cages at their source. If successful alloparenting in wild mice requires simultaneous immigration by related females, the condition would not have been met by any trio mating in this study. However, since inbred mice are identical at their major and minor histocompatibility complexes, one way by which mice identify each other and choose mates [24], there may be other factors at work by which inbred mice identify each other. The histocompatibility alleles of outbred mice are limited, but do differ among individuals in outbred mouse populations, so the females chosen as part of a mated trio would have definitely been socially unfamiliar and may also not have perceived each other as related. The mechanism of reproductive suppression in trio matings is not immediately apparent. Virgin adult female domesticated *Mus* are generally maternal [25, 26], unlike their wild counterparts [27]. Since no infanticide was seen, unlike in wild mice, female competition through litter killing is unlikely. Pups may die for other reasons such as congenital defects [28], and these dead pups may have been missed in the bedding at cage change or completely consumed by the parents. A possible mechanism of reproductive suppression in the female dyad in a trio mating is one female limiting access to the male (mate guarding). This occurs in other species, such as humans, and in the context of the mouse cage, there is limited opportunity for the male mouse to escape a mate-guarding female. However, this explanation would predict a positive correlation between dominance and reproductive success, which we did not find. This suggests that scramble competition or some other non-dominance related mechanism is in play, which would be consistent with the universal presence of alloparenting in laboratory mice. For example, perhaps one female simply wins the reproductive race by being in estrus when mated and her pregnancy and lactation suppresses the estrous cycle of the other. If scramble competition is in play, the most likely mechanism of reproductive suppression in trio mating is through either estrous cycle suppression or early abortion. Reproductive suppression through infanticide would be a welfare issue for trio-mated cages, but welfare concerns associated with other means of reproductive suppression are unknown.

In the trio matings, the female of a particular coat color was not consistently reproductively suppressed in every cage, indicating that the albino males in this case did not show a preference for animals of the same color. Furthermore, this test system showed that the use of coat color is a practical way to easily determine maternal parentage, which will make future study of this and similar phenomena simpler and far less expensive. In fact, as inbred mice are essentially genetically identical, genetic techniques cannot be used to reliably determine parentage. Thus, this technique allows us to study parentage in mice where it may not have been possible before. There has been a resurgence of interest in ‘old school’ coat color genetics in recent mouse ethology and welfare work [29] and this technique is another practical application, limited by only the presence of combinations of dominant and recessive coat color alleles (agouti/black or any pigment/albino) in a particular strain or stock of interest.

When overall production of both individual mice and cage units was examined, reproductive suppression of one female was seen in trio cages. The per-cage reproductive performance of trios and pairs was not significantly different. This might be because there truly is no advantage to breeding in trios (or even that trios in reality perform worse than pairs). Alternatively, the small but non-significant difference between per-cage output of pairs and trios might reflect a biological reality, in which case mating mice in trios may only show statistically detectable benefits when there are larger numbers of breeding mice in a colony. Using a post-hoc power assessment, assuming that the effect size observed in this experiment is the biological reality, to see a statistically detectable benefit to trio mating in 80% of cases, the colony would have to comprise at least 96 cages. However, even if there is a small benefit in pups produced to trio breeding in large colonies, this might never be economically viable as the costs of feeding and handling an extra non-reproductive female, and culling unwanted males (to maintain a 2:1 sex ratio), might easily outweigh the benefit of minor increases in cage-level production.

The difference in nest scores between CF1 and B6, and pairs and trios, is most likely related to the size and number of animals in the cage. CF1 are larger than B6, so it is logical that they would have less thermal stress and therefore would need to build less complex nests [30]. In trio matings, three animals are in the cage generating body heat, thus also mitigating cold stress. The presence of three animals in the cage, however, may also result in lowered nest scores due to mechanical disruption of the nest.

The difference in pup alopecia found between pairs and trios may be due to the fact that there are more animals in a cage and therefore more potential hair-pullers. Alternatively, it could indicate stress or frustration in the adults present in trio cages. Alopecia in adult animals has been examined in the literature [31–34] but very little work has been done on alopecia of nursing mice [35, 36]. For a full review of the ethology and presentation of barbering in mice, please see Dufour and Garner [34]. Since the pups in this study were missing fur from their torsos, but the head was spared, it would seem that one or more of the parents were pulling fur from portions of the offspring easily accessible whilst nursing. Wounds associated with agonistic encounters were not seen on animals in any breeding configuration and no fighting was seen in any cage. As these were stable breeding cages, this is not surprising.

It should be noted that animals tested in this case were healthy mice with no genetic modifications and that all mice were provided with nesting material. Although no benefits in pup weaning weight or survival to weaning were seen with an extra female in this case, animals with genetic modifications that render them smaller or weaker might benefit from alloparenting care or from the thermal benefits of another adult in the cage, if no nesting material or other enrichment is provided. The effectiveness of harem mating, where one male is mated with 3–4 females at a time, and the females are removed and replaced as they become visibly pregnant, was not examined in this experiment. Although this has the potential to greatly increase the output of males, there are benefits to young mice of being reared by both parents, such as timely reproductive development of young females and normalization of behavior [37–39]. Using harem mating or male rotation has other potentially deleterious effects if there are only a small number of males available in that there is the potential for a founder effect to occur and to therefore accelerate drift away from a standard genetic background.

Most importantly, this paper offers a cautionary tale, not just for mouse breeding, but for captive animal breeding in general. First, just because things have always been done one way, or there is received wisdom used to support a practice, does not mean that it is correct. There was enormous outcry over the new cage space recommendations during the comment period for the latest edition of the *Guide for the Care and Use of Laboratory Animals* because many leaders in the field maintained that the new recommendations would be problematic for breeders and scientists alike because it would prevent the use of the more efficient trio mating system. As we have shown here, trio mating is not necessarily the most efficient way to mate mice. Second, just because a behavior is seen in the wild does not mean that it will be expressed in laboratory animals, domesticated animals, or in captive breeding programs—either because the correct stimuli are not present or because it has been selected against. In this case, the existing wild mouse literature clearly predicted that alloparenting should not have occurred. Third, the two senior authors held conflicting views on the value of trio matings and the occurrence of alloparenting in laboratory mice; both were incorrect. Evidence-based decision making should always trump received wisdom and teamwork should always be used to resolve these disputes.

The original questions asked by the investigators were answered; yes, laboratory mice alloparent; but no, there is no advantage to trio mating. The way the animals were mated was reflective of what typically happens in labs, where animals are placed together without regard to estrous cycle, mate choice, or familiarity of paired females in a trio. Having animals enter into a mating cage with either naturally or chemically synchronized estrous cycles might have allowed for more litters to be born at the same time, thus better illustrating alloparenting. Perhaps our more realistic experimental design was not ideal for documenting alloparenting but it did allow for other effects, such as reproductive suppression in trio matings, to emerge. This study shows that for standard laboratory mice provided with an adequate amount of nesting material, production per female is maximized if mice are mated in pairs. Small gains in pup number may be seen in trio matings, but these are likely to be offset by other factors as outlined above. Alloparenting behavior in laboratory mice might be influenced by: relatedness of females; whether females are introduced to the cage simultaneously or sequentially; whether females are introduced to the cage first and allowed to become familiar with each other before a male is introduced; female choice of nursing partners [40]; mate choice [41]; the sex of offspring being alloparented, or other as yet undetermined factors. Further study is needed, since the current work raises more questions than it answers about alloparenting and reproductive suppression in laboratory mice.

## Supporting Information

S1 Checklist ARRIVE Guidelines checklist. (PDF)

S1 Data All data collected from the study. (XLSX)

S1 File The scoring paradigms used to assign numeric scores to alopecia, wounding, and nest complexity. (PDF)
