# Supplementary material for: Two of a Kind or a Full House? Reproductive Suppression and Alloparenting in Laboratory Mice
Source: PLoS One. 2016 May 5;11(5):e0154966. doi: 10.1371/journal.pone.0154966 (PMC4858245; doi:10.1371/journal.pone.0154966)
Supplement: S1 File — (PDF) [file pone.0154966.s003.pdf]

Scoring scales used

Alopecia scoring:

Hair loss can include trimmed whiskers, nude muzzles, or hair missing anywhere on the face. Estimate the overall hair loss from the body including both dorsum and ventrum during cage change.

**0**: Full coat; no observed hair loss
**1**: up to 25% hair loss
**2**: 25-50% hair loss
**3**: 50-75% hair loss
**4**: more than 75% hair loss

Wound scoring

Examine the body of the animal, including both dorsum and ventrum.

**0**: No wounds
**1**: Mild; area affected < $1cm^2$ with bruising or bites. No veterinary care required.
**2**: Moderate; area affected > $1cm^2$ where the majority of wounds are on the surface and do not go through all layers of skin. Veterinary care may be indicated.
**3**: Severe; area affected > $1cm^2$ where the majority of wounds are severe ulcerations and penetrate the skin deeply. Veterinary care definitely required; consider euthanasia

Nest scoring

See Hess, S. E. *et al*. Home improvement: C57BL/6J mice given more naturalistic nesting materials make better nests. *J. Am. Assoc. Lab. Anim. Sci*. 47, 25-31 (2008).

**0**: Undisturbed; no sign of interaction with nesting material
**1**: Disturbed; interaction with nesting material is evident
**2**: Flat; nesting material has been gathered to form a nest site in the cage, but no walls evident
**3**: Cup; nesting material has been gathered into a shallow cup or bowl
**4:** Incomplete dome; nesting material has been gathered and built into a nest with walls reaching approximately half way up an imaginary sphere filling the nest site
**5:** Full dome; nesting material has been gathered to a nest site that is completely enclosed with only a small mouse-sized exit at the top or on the side.
